# Impact of a daily supportive text message program (text4hope) on the stress, anxiety, and depression levels of elderly (60 years and above) subscribers during the COVID-19 pandemic in Alberta.

**DOI:** 10.1192/j.eurpsy.2024.350

**Published:** 2024-08-27

**Authors:** E. Owusu, R. Shalaby, B. Agyapong, W. Vuong, A. Gusnowski, S. Surood, A. J. Greenshaw, V. I. Agyapong

**Affiliations:** ^1^Psychiatry, University of Alberta; ^2^Psychiatry, Alberta Health Services, Edmonton; ^3^Psychiatry, Dalhousie University, Halifax, Canada

## Abstract

**Introduction:**

One of the biggest global crises in our generation is the COVID-19 pandemic. It has had a severe and far-reaching negative impact on our health systems, economies, and societies. Older adults were particularly at higher risk of severe illness, isolation from social distancing measures, and concerns about their health.

**Objectives:**

The objective of this study is to evaluate the impact of the daily supportive text message program (Text4Hope) on the levels of stress, anxiety, and depression experienced by elderly subscribers during the COVID-19 pandemic in Alberta six weeks after enrollment.

**Methods:**

An online survey link was used to gather demographic and clinical information on several self-report scales, such as the Perceived Stress Scale (PSS) ≥ 14 and Generalized Anxiety Disorder 7-item (GAD-7). Scale ≥ 10, and Patient Health Questionnaire-9 (PHQ-9) ≥ 10. Descriptive and inferential statistics were run using SPSS version 25.

**Results:**

172 subscribers out of 1136 completed baseline and six weeks using an online questionnaire, giving a response rate of 15.1%. There were significant reductions in mean scores on the PSS-10 and GAD-7 scales at six weeks compared to baseline (P>.05), but not on the PHQ-9 scale. There were also significant reductions in the prevalence of moderate or high stress (68.6% vs 60.5%, p=0.036) and likely GAD (14.9% vs 22.7%, p=0.029) from baseline to six weeks, with the highest reduction in stress (8.1%). A change (27.6% to 25.2%) in the prevalence of likely MDD from baseline to six weeks was insignificant. (P>.05)

**Conclusions:**

This study’s findings show a decrease in the prevalence rates and the mean scores for stress and anxiety on standardized scales, indicating an improvement from baseline to six weeks. This outcome has potential implications for planning an intervention to meet the mental health needs of the elderly in similar situations like the pandemic

**Disclosure of Interest:**

None Declared

